# Crowding can impact both low and high contrast visual acuity measurements

**DOI:** 10.1038/s41598-022-20479-y

**Published:** 2022-09-29

**Authors:** František Pluháček, John Siderov, Ivana Macháčová

**Affiliations:** 1 Department of Optics, Palacký University Olomouc, Olomouc, Czech Republic; 2 Centre for Vision Across the Life Span, Department of Optometry and Vision Sciences, University of Huddersfield, Huddersfield, HD1 3DH UK

**Keywords:** Visual system, Vision disorders

## Abstract

The adverse impact of adjacent contours on letter visual acuity is known as crowding but there is conflicting evidence that foveal crowding may be reduced or disappears under low contrast conditions. Potential differences in foveal crowding with contrast on clinical measurements of visual acuity, including test–retest repeatability, were assessed. Visual acuity was measured at the fovea on adult participants with normal vision under three different contrast levels (− 90, − 10 and − 5%). Three rows of 5 letters, each row differing in size by 0.05 logarithm of the minimum angle of resolution (logMAR) from largest to smallest were displayed at the center of a monitor. Crowding was varied by varying the separation between horizontally adjacent letters from 100% optotype size to 50%, 20% and 10% optotype size. Inter-row spacing was proportional to optotype size. Observers read the letters on the middle row only. Measurements continued by reducing the size of the letters until 3 or more errors were made and were repeated on two separate days. Visual acuity worsened as both letter contrast decreased and inter-optotype separation reduced (expressed as a percentage of letter width). When expressed in minutes of arc of separation the impact of crowding was the same across all contrasts. Crowding occurs for both high and low contrast charts and should be considered when assessing low contrast visual acuity. Test–retest repeatability showed little or no dependence on either contrast or inter-optotype separation.

## Introduction

High contrast visual acuity (VA) measurements are a key part of a routine ophthalmic examination^1^, and used to inform clinical decision making for different ocular abnormalities such as the correction of refractive error^2^ or the management of patients with cataract^3^. Although the use of high contrast VA charts is ubiquitous in ophthalmic settings, the measurement of low contrast acuity to assess functional visual ability for patients with normal vision, or reduced vision due to ocular disease has been advocated for some time^4,5^. Low contrast letter charts also have an important role in assessing patients with neurological conditions such as multiple sclerosis where functional vision loss cannot be detected using high contrast charts^6^.

Visual acuity reduces in a non-monotonic fashion as optotype contrast decreases^7^. On a standard Bailey–Lovie logMAR chart, this reduction is equal to about a 2.5-line difference from 8% compared to 90% contrast visual acuity^4^. Several different types of low contrast letter charts have been recommended. Some like the Pelli–Robson chart^8^, are constructed using letters of the same size but of different contrasts. Others are based on the Early Treatment Diabetic Retinopathy Study (ETDRS) logarithm of the minimum angle of resolution (logMAR) format^9^, comprising letters in rows of decreasing size on charts with different contrast levels^10^. In both formats of low contrast letter charts, the separation between adjacent letters and rows of letters is controlled. Either a fixed separation of one letter width as in the Pelli–Robson chart or varying as a fixed proportion of the size of the letters in the line directly below, for ETDRS based formats^10^. The relative separation of letters and rows of letters in the ETDRS chart format also provides a consistent crowding effect, defined as the detrimental impact on visual acuity arising from the presence of adjacent letters^11^.

Although the focus of the present study is on clinical measurements of foveal letter acuity, crowding can also be demonstrated in other spatial vision tasks including measurements of Vernier acuity^12–15^, stereoacuity^16–18^ and others^19,20^.

Crowding is strongest in the retinal periphery^19^, nevertheless, crowding is an important consideration when measuring foveal visual acuity, particularly when measuring visual acuity in children or in patients with amblyopia^11,21–24^. For high contrast optotypes and unlimited viewing, the spatial extent of foveal crowding has been quantified and shown to extend in normal observers over a relatively short distance of a few minutes of arc, equivalent to about one letter size at threshold^25–30^. However, for low contrast optotypes, some investigators have reported that crowding is either absent or minimal in foveal vision^31–34^. These findings contrast with several studies that have reported strong crowding effects using low contrast targets in the periphery^35–37^. This discrepancy may be explained at least in part, on the basis of laboratory experiments that showed, for unlimited viewing conditions, the spatial extent of foveal crowding is fixed, irrespective of target contrast, and occurs over only a few minutes of arc^38–40^. Thus, for crowding to impact on measurements of low contrast foveal visual acuity, crowding flankers would need to be placed within a few minutes of arc of the acuity target rather than as multiples of the target’s stroke width or size as many previous studies have done^31–34^, as the resultant inter-optotype separations fall outside of the critical crowding distance. Recent studies^41,42^ have also shown that crowding depends on the viewing time, if the time is very brief (typically less than 240 ms)—the shorter the time the stronger crowding effect. Crowding may therefore become more evident with very brief exposure durations, although clinical measurements of visual acuity are typically taken with unlimited viewing times.

For clinical measurements of low contrast acuity therefore, the impact of crowding may vary depending on both the contrast of the optotypes and their inter-optotype separation. Given the importance of low contrast visual acuity measurements, and the reported reduction in crowding under low contrast conditions, we investigated whether crowding affects clinical measurements of low contrast visual acuity, including test–retest repeatability by carefully varying the inter-optotype separation for a range of contrast conditions.

## Results

Unsurprisingly, visual acuity was reduced as contrast was decreased. On average, across all inter-optotype separations and test–retest conditions, intermediate and low contrast logMAR was 0.21 and 0.32 higher than the average high-contrast logMAR, respectively. Table 1 presents a summary of the measurements (mean logMAR and standard deviations) for each inter-optotype separation and contrast condition.

**Table 1:** Mean logMAR for the first, *VA*_1_ and second, *VA*_2_ measurements of visual acuity and their standard deviations SD_*VA*1_, SD_*VA*2_ respectively, under all contrasts and letter separations tested.

Weber contrast	− 90%				− 10%				− 5%			
Separation (% of letter width)	10	20	50	100	10	20	50	100	10	20	50	100
*VA*_1_	− 0.004	− 0.061	− 0.131	− 0.182	0.177	0.136	0.068	0.068	0.278	0.237	0.184	0.196
SD_*VA*1_	0.077	0.082	0.083	0.110	0.074	0.086	0.097	0.097	0.093	0.101	0.104	0.095
*VA*_2_	− 0.008	− 0.066	− 0.139	− 0.194	0.181	0.124	0.066	0.049	0.264	0.231	0.174	0.189
SD_*VA*2_	0.069	0.071	0.086	0.085	0.099	0.087	0.097	0.116	0.095	0.086	0.111	0.110

**Figure 1:**
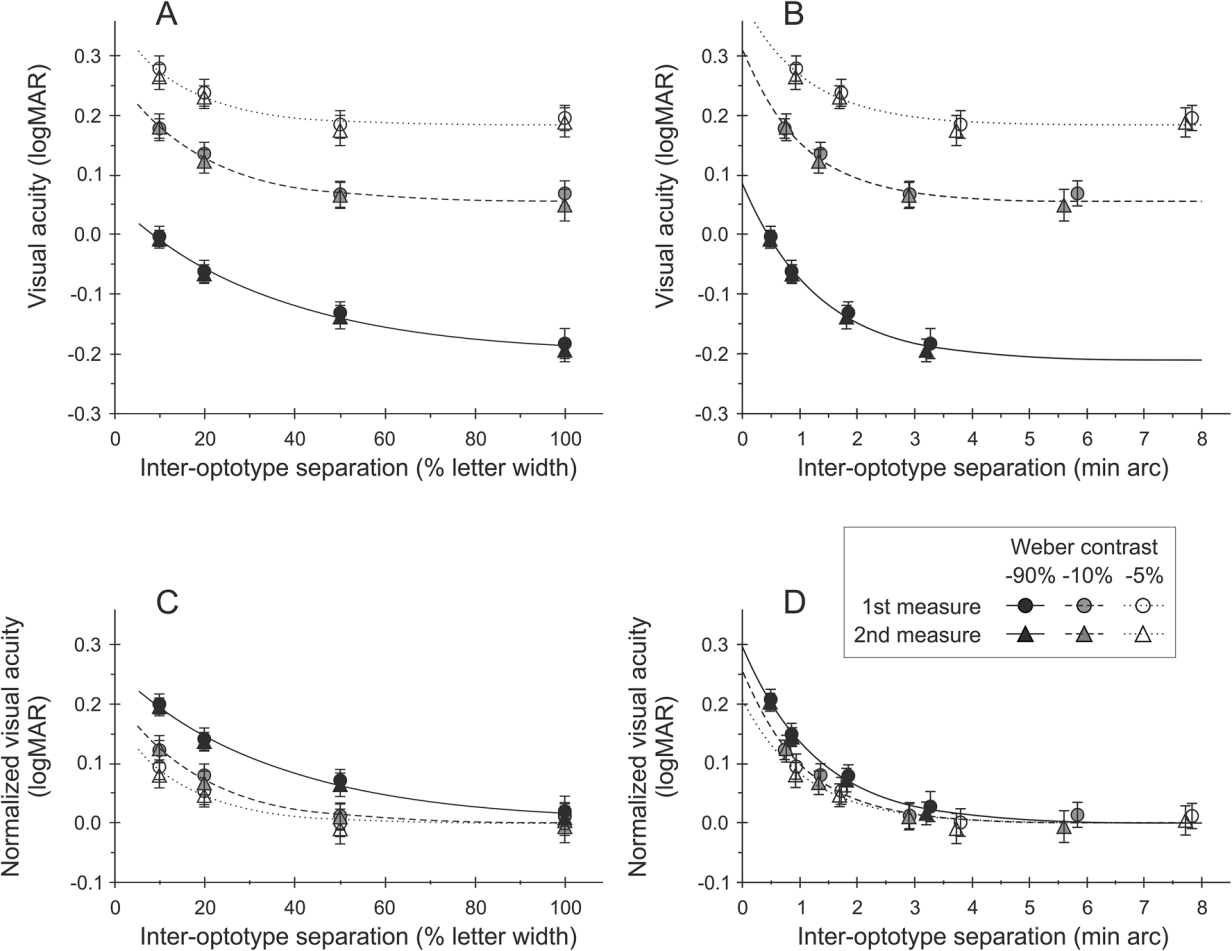
The top left panel (**A**) shows the average logMAR across all subjects, plotted against edge-to-edge inter-optotype separation (% letter width) for high (open symbols), intermediate (grey symbols) and low (closed symbols) contrast levels. Data for test (circles) and re-test (triangles) are shown. The data set for each contrast condition is fitted by an exponential curve. The top right panel (**B**) shows the same data, but this time plotted against inter-optotype separation in min arc. The bottom panels (**C**) and (**D**) plot logMAR acuity, normalized to the asymptotic value of each exponential curve. It is evident in all 4 panels that logMAR increases both as a function of decreased contrast and reduced inter-optotype separation. In the two left panels (**A**) and (**C**), the increase appears less under the intermediate and low contrast conditions. However, this difference is much less evident in the two righthand panels (**B**) and (**D**), in which the letter separation is expressed in min arc. An example showing the results from an individual subject is shown as a Supplementary File.

Figure 1 plots the average logMAR across all observers as a function of the inter-optotype separation (percent letter width) (panel A) and min arc (panel B). The closed (filled) symbols represent the high contrast condition, the shaded (grey) and open symbols represent the intermediate and low contrast conditions, respectively. The first, test measurements are shown by the circles and the second, retest measurements by the triangles. The error bars represent ± 1 standard error (SE). The data sets for each contrast condition were fit by the exponential curve described in the methods [Eq. (1)]. The bottom two panels (C and D) show the same averaged data but normalised by subtraction of the term *VA*_0_ of the respective fitted exponential curves, so all the re-fitted exponential curves converged to a visual acuity of zero (i.e., *VA*_0_ = 0). The normalised plots provide a helpful comparison of the shape of the studied dependences.

Figure 1 shows that logMAR increases as both inter-optotype separation and level of contrast decreases, irrespective of the way in which inter-optotype separation is recorded. However, the increase in logMAR (i.e., poorer visual acuity) for the closer inter-optotype separations, is more marked (by about 0.5 logMAR normalized) for the high contrast condition compared to the intermediate or low contrast conditions, if the inter-optotype separation is expressed in terms of percent letter width (compare panels C and D in Fig. 1). Under the closest inter-optotype separation (10% letter width) high contrast logMAR increases approximately by one and a half times as much as the comparable inter-optotype separation condition under the intermediate contrast condition and more than twice as much as under the low contrast condition (see Fig. 1, panel C with normalised data). This is supported by the outcome of the two-way repeated measures ANOVAs, which revealed significant effects of separation (*F*_3, 60_ = 134.426, *p* < 0.0001 and *F*_3, 60_ = 124.487, *p* < 0.0001), contrast (*F*_1.31, 26.26_ = 327.293, *p* < 0.0001 and *F*_1.63, 32.70_ = 380.071, *p* < 0.0001) and a significant separation x contrast interaction (*F*_6, 120_ = 7.198, *p* < 0.0001 and *F*_6, 120_ = 9.053, *p* < 0.0001) for both test, and retest measurements, respectively.

On the contrary, if the inter-optotype separation is expressed in min arc, the shapes of the logMAR curves are similar for all contrast conditions and the critical angular separation at which crowding begins to reduce visual acuity appears to be essentially constant (see Fig. 1, panel D with normalised data). To support this statement statistically, the dependence of visual acuity (logMAR) on separation (min arc) for each subject under all tested contrasts was fit by an exponential curve [see Eq. (1)] as described in the “Data analysis” section. The shape of the curve (steepness) is determined by *σ*, while *s*_0_ and *VA*_0_ change its horizontal and vertical positions. The numerical iterative fitting algorithm did not converge properly for four subjects and the respective results showed large over-estimations (e.g., over-estimates of *σ* were 143-, 264-, 282- and 453-min arc while the average of all other values was 1.5 ± 1.7 with a maximum value of 11). So, the data of these four subjects were not included in the subsequent statistical analysis. The resulting one-way repeated measures ANOVAs revealed that the parameters *σ* and *s*_0_ did not differ significantly between different contrasts (*F*_1.49, 23.87_ = 0.703, *p* = 0.465 and *F*_2, 32_ = 0.112, *p* = 0.895), if inter-optotype separations were considered in terms of min arc. However, the changes of *VA*_0_ were significant (*F*_1.182, 18.917_ = 104.789, *p* < 0.0001). As can be seen in the panel D of Fig. 1, the apparently greater magnitude of crowding for high contrast letters (i.e., in Table 1 and Fig. 1A or C) can be attributed to the smaller letter-to-letter separations, in min arc, between neighbouring high-contrast letters compared to between the medium- and low-contrast letters. Thus, the decrease in contrast caused a systematic reduction in visual acuity (represented by the increase of *VA*_0_ in terms of logMAR), which was independent of angular separation, i.e., the magnitude and extent of the crowding effect was the same irrespective of the contrast.

**Figure 2:**
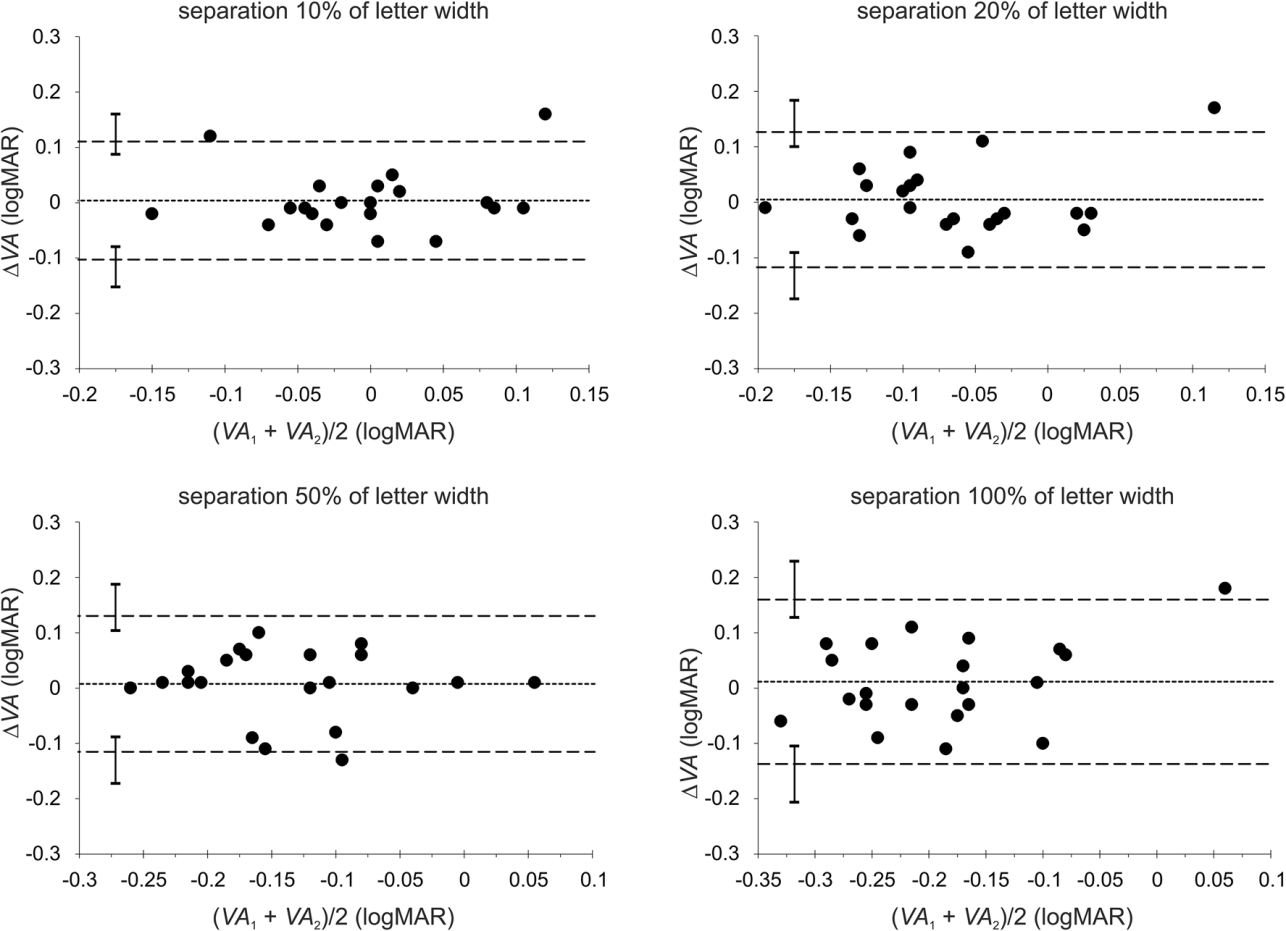
Bland and Altman^43^ plots depicting the difference ∆*VA* in logMAR between the first *VA*_1_ (test) and second *VA*_2_ (retest) measurements against their average for the high (− 90%) contrast level. The dashed lines represent the 95% limits of agreement (LoA) and the dotted lines the mean differences. The error bars shown in each panel represent the 95% confidence intervals for the Bland–Altman LoAs^44^.

**Figure 3:**
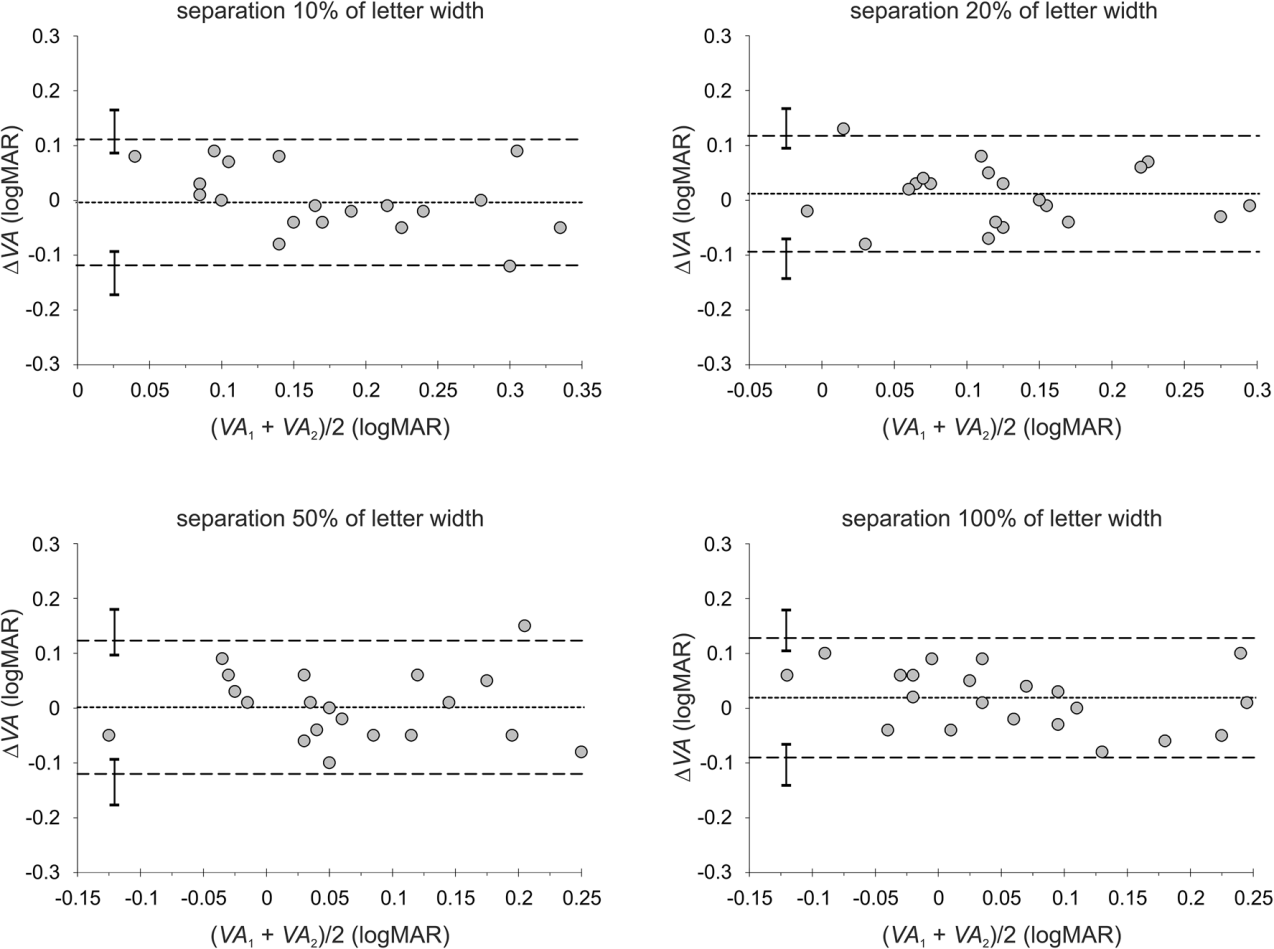
Bland and Altman^43^ plots depicting the difference ∆*VA* in logMAR between the first *VA*_1_ (test) and second *VA*_2_ (retest) measurements against their average for the intermediate (− 10%) contrast level. The dashed lines represent the 95% limits of agreement (LoA) and the dotted lines the mean differences. The error bars shown in each panel represent the 95% confidence intervals for the Bland–Altman LoAs^44^.

**Figure 4:**
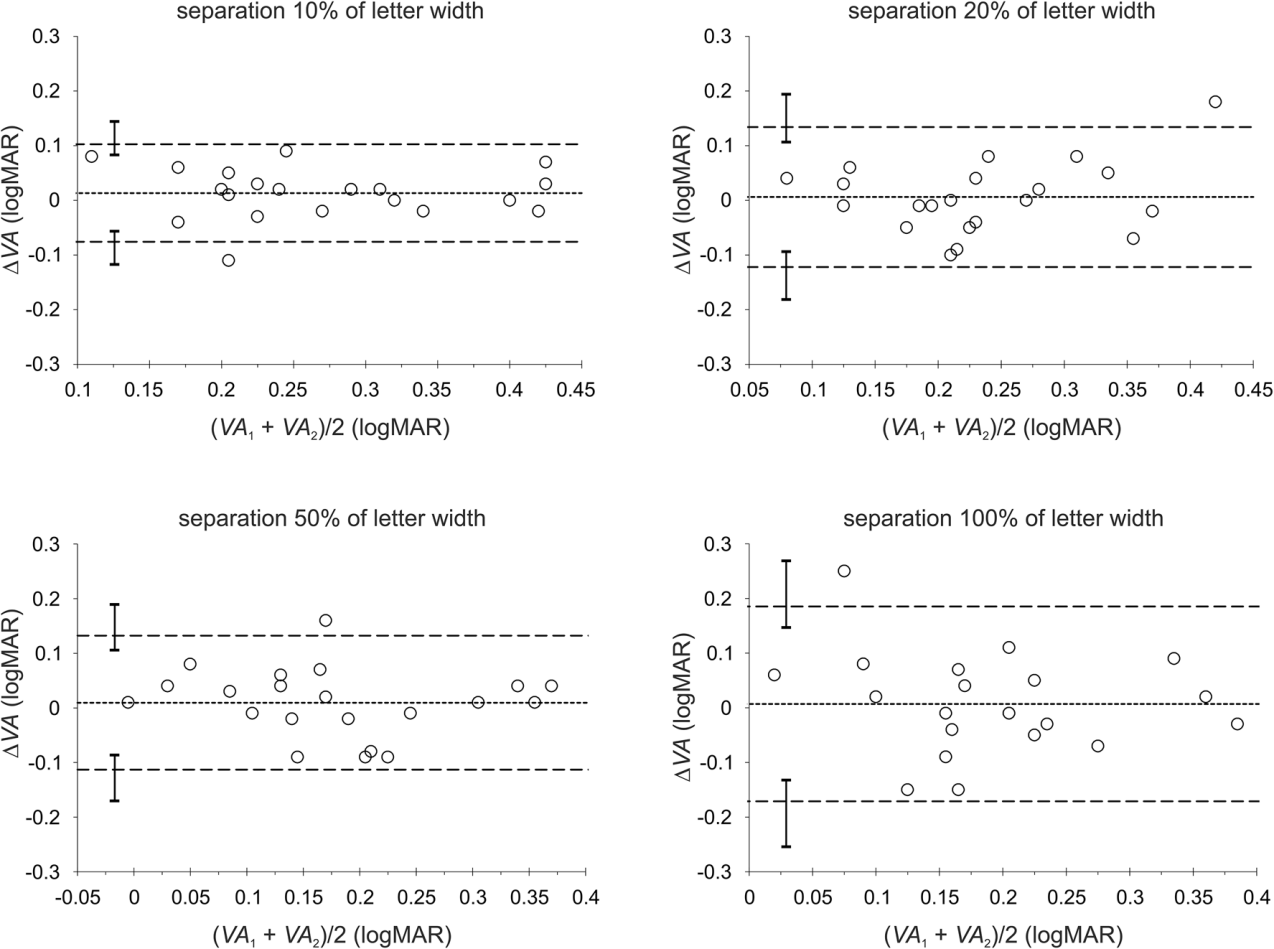
Bland and Altman^43^ plots depicting the difference ∆*VA* in logMAR between the first *VA*_1_ (test) and second *VA*_2_ (retest) measurements against their average for the low (− 5%) contrast level. The dashed lines represent the 95% limits of agreement (LoA) and the dotted lines the mean differences. The error bars shown in each panel represent the 95% confidence intervals for the Bland–Altman LoAs^44^.

A summary of the test–retest repeatability measurements is presented in Table 2. Shown are the mean differences (∆*VA*) in visual acuities of test (*VA*_1_) and retest (*VA*_2_) measurements, the associated standard deviations, and the relevant coefficients of repeatability (CoRs) for each condition of contrast and optotype separation. The corresponding Bland–Altman plots^43^ for each contrast condition with the associated limits of agreement (LoAs) are presented in Figs. 2, 3, and 4 for each of the four inter-optotype separations. In each panel of Figs. 2, 3, and 4, the difference in logMAR between test and retest measurements is plotted against the average logMAR across test and retest for each inter-optotype separation condition. Dashed lines represent the 95% LoAs and the dotted lines the mean differences. The error bars shown in each panel represent the 95% confidence intervals for the Bland–Altman LoAs^44^. Mean differences between test and retest measurements were statistically insignificant for all conditions tested (*p* > 0.13, paired t-test).

**Table 2:** Mean differences, Δ*VA* (logMAR) of the test–retest measurements (see Table 1), the associated standard deviations SD_Δ*VA*_, and coefficients of repeatability CoRs (1.96 × SD_Δ*VA*_) under all contrasts and letter separations tested.

Weber contrast	− 90%				− 10%				− 5%			
Separation (% of letter width)	10	20	50	100	10	20	50	100	10	20	50	100
Δ*VA*	0.004	0.005	0.008	0.011	− 0.004	0.012	0.001	0.019	0.013	0.006	0.010	0.007
SD_Δ*VA*_	0.054	0.062	0.063	0.076	0.059	0.054	0.062	0.056	0.046	0.065	0.063	0.091
CoR	0.107	0.122	0.123	0.149	0.115	0.106	0.122	0.109	0.089	0.128	0.123	0.178

The calculated 95% CI for the LoAs show that the distributions of LoAs between separations for given contrast levels were similar (i.e. the error bars overlapped) with the exception of the 10% and 100% inter-optotype separations for the low contrast condition (i.e., the error bars do not overlap) (Fig. 4). Thus test–retest repeatability exhibits no significant dependence on either the contrast or inter-optotype separation except for the low contrast level. This statement is also supported by values of CoRs, which differ by less than about 0.04 (Table 2) between contrast conditions as well as between separation conditions for a given contrast, except the low contrast condition, where the CoR for the 10% separation is approximately half compared with the 100% separation condition (Table 2).

## Discussion

Consistent with previous reports, visual acuity (logMAR) tested foveally using low contrast letter charts was considerably worse than when tested under high contrast conditions^4,7,45,46^. On average, across all test conditions (i.e., all inter-optotype separations and test and retest measurements), there was a worsening of 0.21 logMAR from the high (− 90%) to intermediate (− 10%) contrast conditions and a further increase of 0.11 logMAR to the lowest (− 5%) contrast condition.

In contrast with previous studies that have shown little or no effect of crowding with low contrast optotypes^31–34^, a robust foveal crowding effect was found in the present study across all of the contrasts tested. When the inter-optotype separation was expressed in terms of percent letter width, the results suggest that crowding is stronger for the high contrast condition (Fig. 1C with normalised data) albeit still present for close inter-optotype separations. However, a key difference between the present study and previous results at the fovea is that when inter-optotype separation is plotted as a function of the angular separation in min arc, high, intermediate, and low contrast letter acuities were affected similarly as inter-optotype separation was reduced (i.e., as crowding increased). This finding is clearly shown by the similarity in the magnitude of the crowding effect across contrasts (Fig. 1D with normalised data). The present clinical results are also consistent with previous laboratory reports that showed the spatial extent of foveal crowding is limited to a short distance of only a few minutes of arc, irrespective of target contrast^38,39^. Previous studies using low contrast acuity targets used inter-optotype separations based on the proportion of optotype size that placed the flanking letters at or beyond the critical crowding distance (e.g.^31–33^). An exception was the report by Bailey and Raasch^31^ who addressed a similar question as the present study and reported their results both in terms of inter-optotype separations based on letter width and angular separation in min arc. However, the smallest angular separation they used was 2 min arc which results in only a relatively small crowding effect, if any at all (see Fig. 1D).

Test–retest repeatability as expressed in terms of the CoR ranged from 0.089 to 0.178 across all inter-optotype separations and contrast conditions. Unlike some previous studies^47,48^, a consistent reduction in test–retest repeatability as contrast decreased was not found (see Table 2). For high contrast letters at the standard ETDRS inter-optotype separation (100% optotype width), repeatability was approximately ± 0.15 logMAR, similar to some previous reports of high contrast test–retest repeatability using Bailey–Lovie or ETDRS type formats^48,49^, although not as good as others^3,47^. For intermediate (− 10%) and low (− 5%) contrast letters at the standard ETDRS inter-optotype separation (100% optotype width), test–retest repeatability varied from about ± 0.11 logMAR to ± 0.18 logMAR, respectively. These results approximate previous reports of repeatability using low contrast optotypes^45,47^. Potential differences with other studies may reflect differences in the step size we used between rows (0.05 logMAR) or differences in the level of contrast.

There have been suggestions that measurements of visual acuity using more crowded charts could improve the reliability of measurements^50^, presumably through a process whereby crowding steepens the slopes of the underlying psychometric functions for visual acuity measurements^22^. If this were true, we would have expected to find better test–retest repeatability for the closer inter-optotype separations where crowding was stronger. However, inter-optotype separation (i.e., crowding) had little or inconsistent effect on test–retest repeatability. For the low contrast condition, the CoR at the closest (10% optotype width) separation was about half as much as that of the largest (100% optotype width) separation (Table 2). A similar trend was also seen for the high contrast condition, albeit not as marked, but not for the intermediate contrast. Thus, at least for observers with normal vision, crowding does not improve reliability of foveal acuity measurements.

Our results describe new findings that show foveal crowding occurs for clinical measurements of low contrast visual acuity, consistent with previous experimental results^38,39,51^. Our results are also consistent with the effects of contrast on crowding in peripheral vision^52–54^. For clinical measurements of low contrast visual acuity using standard ETDRS charts or similar where the inter-optotype separation is fixed at 1 letter width (100% optotype) and with unlimited viewing, similar to high contrast conditions, crowding does not occur. However, where the separation between target letters and flankers is reduced, for example when measuring low contrast visual acuity in children^33^ or where the extent of crowding may be larger^24^, the impact of crowding will be more relevant. Where letter targets are shown with brief presentation times (i.e., less than 240 ms), crowding may become more apparent even with larger letter-to-letter separations^41,42^. Our results may also be important when assessing low contrast acuity in pathological conditions where the effects may be quite different.

## Methods

### Subjects

Participants were recruited from the Palacký University community in Olomouc, Czech Republic. In total 21 adult participants took part in the study (4 males and 17 females, age range 19–37 years). This number of participants was sufficient to find an acuity difference between measurements, if it existed, of 0.1 logMAR based on a power of 80% and significance level of 0.05 (paired t-test). Participants self-reported to be free from ophthalmic pathology or any systematic condition known to affect vision and had normal or corrected-to-normal vision of at least 0.0 logMAR (6/6). The research was conducted in accordance with the tenets of the Declaration of Helsinki, and written informed consent was obtained from each participant before beginning data collection and after all the procedures and risks were explained. The study was approved by the Ethics Committee of the Faculty of Science of Palacký University Olomouc.

### Stimuli

**Figure 5:**
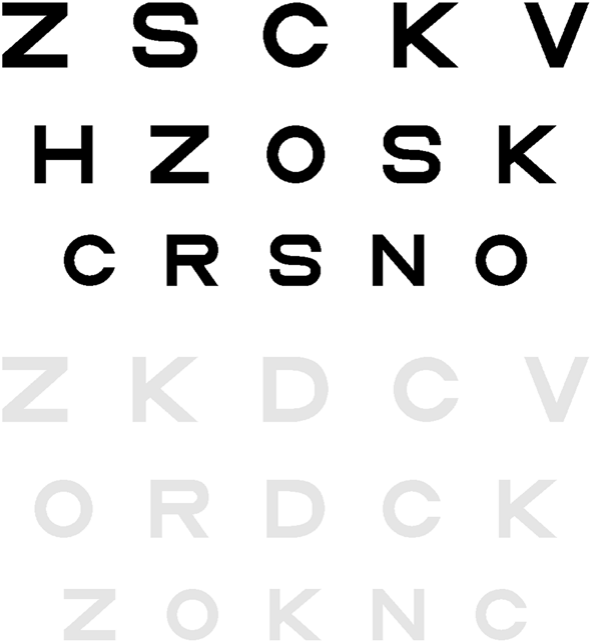
An example of the high (top) and low (bottom) contrast experimental stimuli showing 100% separation between letters. The size of each row of letters differs by 0.05 logMAR.

The experimental set up was similar to one used in a previous study^55^. Briefly, the stimuli consisted of 3 horizontal rows of 5 Sloan letters (C D H K N O R S V Z) of the same size (selected at random but with a constraint that the letters on each row were different), presented at the center of a display monitor. The monitor (ASUS VW 220TE, LCD) measured 56 cm diagonally, with 1680 × 1050 pixel resolution with a background luminance of 228 cd/m^2^. The luminance of the letter stimuli was adjustable to achieve Weber contrasts of − 90% (high), − 10% (intermediate) and − 5% (low) (Fig. 5). Screen luminance was measured using a luminance meter, LMT L1003 (http://www.lmt.de/) under the same ambient illumination used during the experiments. Letter size was decreased from the upper to the lower row in accordance with exponential scaling using a step size of 0.05 logMAR between rows and edge-to-edge letter and row separation was adjustable. The letter stimuli were generated using custom software designed by one of the authors (FP). Exposure duration was unlimited.

### Procedure

The experimental procedures were similar to those used previously^55^. Visual acuity (VA) was measured monocularly with appropriate refractive correction, if required, using the sighting dominant eye of each observer, established using the “hole-in-the-card” method. The non-viewing eye was occluded. Ambient illumination in the experimental room was dim and constant during all measurements. Observers read aloud the letters on the middle row of the stimulus, from left to right (Fig. 5). Guessing was encouraged if the letters were indistinct. Testing began at a letter size at which all letters on the row could be read. The heights of all rows were then reduced in size by a step corresponding to 0.05 logMAR. Testing continued until 3 or more letters were read incorrectly on a row. Final visual acuity (logMAR) was determined using letter-by-letter scoring^56^. Each letter read correctly on each row was scored as 0.01 log units.

The stimuli were viewed with three different Weber contrasts, high (− 90%), intermediate (− 10%) and low (− 5%). The decrease in contrast of the acuity targets was realised by increasing the stimulus luminance while the background luminance of the monitor stayed unchanged. The edge-to-edge, inter-optotype separation of the letters in each row was varied to produce 4 different separation conditions of 100%, 50%, 20% and 10% of the letter width. For each condition, row separation was fixed at the optotype size of the row below. The different inter-optotype separations were presented in random order for each contrast condition tested. To accommodate all five letters on each row, the viewing distances were set at 12 m for logMAR < 0.4 of the central row and 6 m for logMAR ≥ 0.4 of the central row, respectively.

A 2.5 mm pinhole mounted in specially adapted goggles, was placed in front of the tested eye at the spectacle plane to limit the field of view and to reduce optical aberrations, which could affect the results. If a spectacle correction was required, the pinhole was positioned at the center of the ophthalmic lens. The goggles and pinhole were used for all three contrast conditions.

Visual acuity measurements were performed twice (on different days) for each observer and under all conditions to assess repeatability. Before beginning data collection, observers completed practice measurements performed under the high contrast condition using an inter-optotype separation of 100% letter width.

### Data analysis

Visual acuity (logMAR) under different contrasts and inter-optotype separations (in terms of percent letter width) was analysed using two-way repeated-measures ANOVA (factors separation and contrast) with a significance level of 5%, run separately on the test and retest measurements. If required, the levels of statistical significance included a Huynh–Feldt correction for departures from sphericity^57^.

Visual acuity values, *VA* (logMAR), from test and retest were pooled and their dependence on the angular inter-optotype separation, *s,* for each subject and each contrast condition was fit by an exponential curve of the form:

1 $$VA(s) = {e }^{-\frac{s-{s}_{0}}{\sigma }}+{VA}_{0}$$

where *e* is Euler’s number (*e* ≈ 2.718). The curve parameters were determined using a least-squares criterion (Levenberg–Marquardt numerical method). The computations were realised by using STATISTICA 12. The curve parameters obtained for all subjects under different contrasts were analysed using one-way repeated-measures ANOVAs (factor contrast) with a significance level of 5% and when necessary, a Huynh–Feldt correction for departures from sphericity^57^. Each parameter was analysed by separate ANOVAs.

Test–retest repeatability was expressed as the limits of agreement defined as the interval that includes 95% of the measurements (95% LoA = mean difference ± 1.96 × standard deviation of the differences)^43,58^. The 95% confidence intervals for the LoAs were also calculated^44^. The statistical comparison of LoAs was based on the comparison of these intervals (if the intervals overlap, the LoAs did not differ significantly at the level of significancy 5%). Coefficients of repeatability (CoR = 1.96 × standard deviation of the differences) are also reported^59^. Paired t-tests were used to assess differences in the means.

## Supplementary Information

Supplementary Figure S1.

## Data Availability

The datasets used and/or analysed during the current study available from the corresponding author on reasonable request.
