# Supplementary figures and images for: Crowding can impact both low and high contrast visual acuity measurements
Source: Sci Rep. 2022 Sep 29;12:16338. doi: 10.1038/s41598-022-20479-y (PMC9522725; doi:10.1038/s41598-022-20479-y)

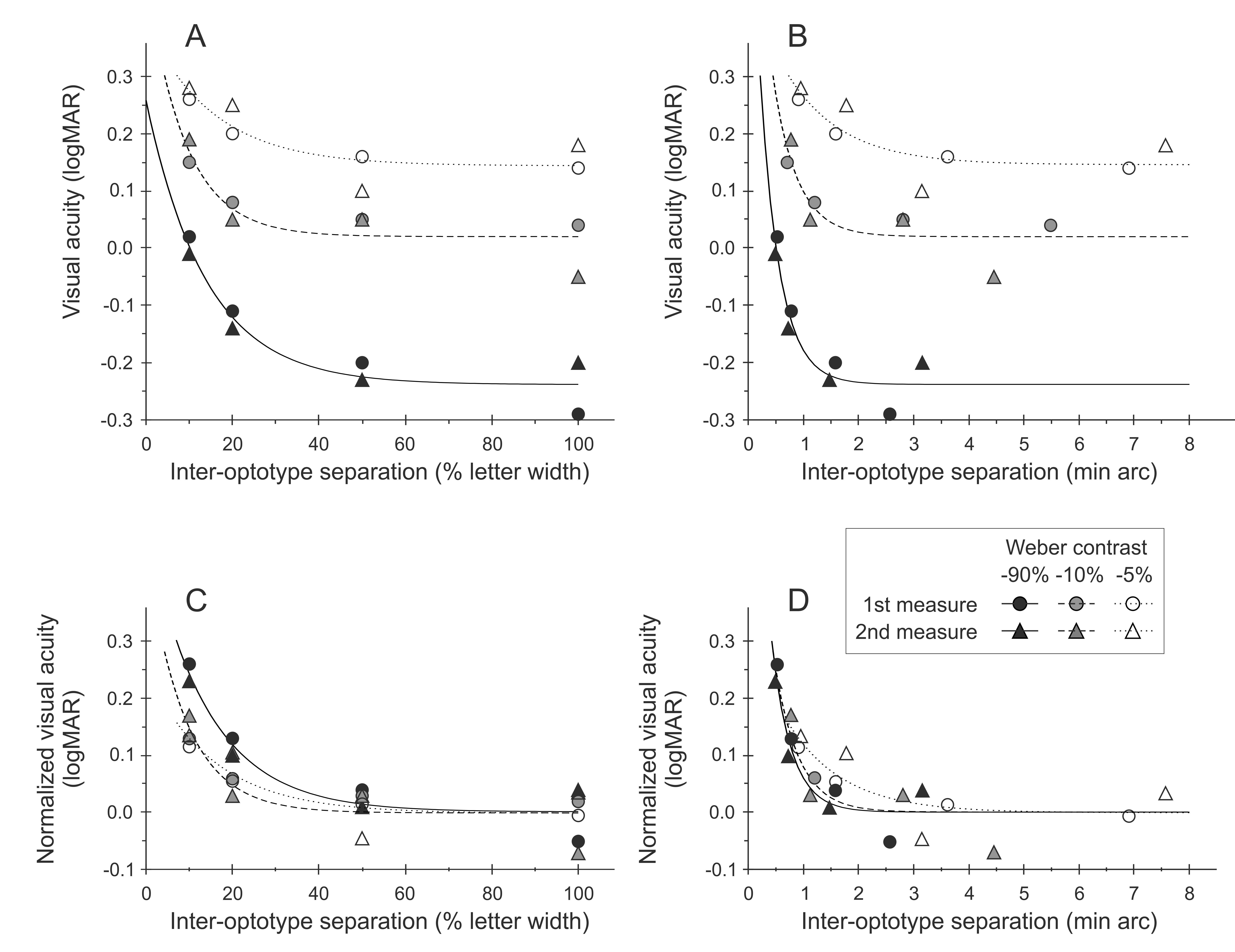

Supplement: Supplementary file 1 — Supplementary Figure S1. [file 41598_2022_20479_MOESM1_ESM.tif]
